# Early Introduction of cART Reverses Brain Aging Pattern in Well-Controlled HIV Infection: A Comparative MR Spectroscopy Study

**DOI:** 10.3389/fnagi.2018.00329

**Published:** 2018-10-18

**Authors:** Jasmina M. Boban, Dusko B. Kozic, Snezana V. Brkic, Dajana F. Lendak, Majda M. Thurnher

**Affiliations:** ^1^Faculty of Medicine, University of Novi Sad, Novi Sad, Serbia; ^2^Center for Diagnostic Imaging, Oncology Institute of Vojvodina, Sremska Kamenica, Serbia; ^3^Clinic for Infectious Diseases, Clinical Center of Vojvodina, Novi Sad, Serbia; ^4^Department of Biomedical Imaging and Image-guided Therapy, Medical University of Vienna, Vienna, Austria

**Keywords:** HIV, brain, MR spectrocopy, aging, HIV infections

## Abstract

**Introduction:** The aim of this study was to compare age-related changes in chronically infected, asymptomatic HIV-positive patients under combination antiretroviral therapy (cART), with age-, gender-, and educational-level-matched healthy subjects, using multi-voxel magnetic-resonance spectroscopy (MRS).

**Methods:** There were 66 chronically infected HIV-positive subjects and 65 age-, gender-, and educational-level-matched control subjects, divided into four groups according to the age: group 1 (20–29 years old), group 2 (30–39), group 3 (40–49) and group 4 (50–59). MRS was performed and ratios of *N*-acetyl-aspartate (NAA)/creatine (Cr) were analyzed in ten locations of the supracallosal gray matter. For the comparison of NAA/Cr ratios in healthy and HIV-positive subjects, ANCOVA with age and education as covariates was performed. Correlations of NAA/Cr ratios with duration of cART were performed using Pearson’s correlation test. Statistical significance was set at *p* < 0.05.

**Results:** The NAA/Cr ratios were decreased in the 20–29-year-old HIV-positive subjects in 8/10 locations (*p* < 0.005) compared to the healthy controls, while in the 50–59-year-old groups they were significiantly lower only in one location (*p* = 0.004). There were significant positive correlations of NAA/Cr levels with the duration of cART in the oldest group of HIV-positive subjects, while in the youngest group there were no significant correlations.

**Conclusion:** The aging pattern in chronic HIV infection under cART is accentuated rather than accelerated. There is an initial HIV-related neuronal damage with a significant decline in NAA/Cr ratios; after the initiation of cART, however, NAA/Cr ratios increase continuously to become similar to healthy aging individuals, probably due to beneficial effect of long-standing cART.

**Summary:** Brain aging in chronic HIV infection under cART is accentuated, with an initial HIV-related neuronal damage followed by a subtle NAA/Cr increase after the initiation of cART. Under cART, in advanced age, NAA/Cr ratios become similar to healthy aging individuals.

## Introduction

The aging pattern in chronic HIV infection has become an important health issue due to increasing percentage of HIV-positive individuals who reach senium with early introduction of combination antiretroviral therapy (cART) (Deeks et al., 2013). There is an ongoing debate about whether a neurocognitive decline in chronic HIV infection represents an accentuation of physiological age-related changes, or an acceleration of otherwise normal brain aging (Sheppard et al., 2017). The results of recent neuropsychological studies in HIV-related cognitive changes have raised the hypothesis of an accelerated brain aging process in chronic HIV infection (Simioni et al., 2010; Shouten et al., 2016).

In normal aging, neuroimaging studies using volumetric and morphometric measurements have shown a loss of gray matter in the prefrontal cortex and a loss of white matter in precentral gyri, the gyrus rectus and the corpus callosum (Harada et al., 2013). In chronic HIV infection, atrophy of almost all brain regions has been reported, predominantly in the frontal and temporal regions, and more prominent than in matching healthy controls (Guha et al., 2016). Age-related decline has also been thoroughly studied with diffusion tensor imaging (DTI), which showed decline in white matter tract integrity in healthy controls (Bennett et al., 2017). However, DTI studies have failed to show any significant differences between healthy and HIV-positive subjects (Watson et al., 2017).

Recent MR spectroscopic studies have demonstrated progressive decline in *N*-acetyl-aspartate (NAA) with relatively stable levels of choline (Cho) and myoinositol (mI) over the years in healthy aging (Eylers et al., 2016). There are only a few single-voxel MR spectroscopic studies that have evaluated the effects of HIV infection in relation to age. A steady decline in the NAA/Cr ratio was observed in the basal ganglia, the frontal and parietal gray matter and the white matter in primarily treated HIV patients, of whom majority presented with signs of HIV-associated dementia (Ernst et al., 2010; Chang et al., 2014). One study also confirmed a steady increase in mI/Cr and Cho/Cr ratios in the white matter in HIV patients on cART (Harezlak et al., 2011). Up to date, to the best of our knowledge, no comparative multivoxel MR spectroscopy studies have been conducted in treated HIV patients with regard to age-related changes. In addition, a majority of the published studies on HIV-related aging were performed with no clear differentiation between demented and non-demented, or treated and therapy-naïve HIV patients, due to small patient samples (Holt et al., 2012).

The aim of this cross-sectional study was to compare age-related changes in chronically infected, asymptomatic HIV-positive patients under long-standing cART, with age-related changes in age-, gender- and educational-level-matched healthy subjects, using two-dimensional (2D) multi-voxel magnetic resonance spectroscopy (MRS).

## Materials and Methods

### Study Population

A group of 121 subjects fulfilled the study inclusion criteria, and underwent a multi-voxel 2D MRS study after signing a fully informed written consent form. The study was approved by the Institutional Ethics Committee board. There were 66 chronically infected HIV-positive subjects, mean age 37.84 ± 7.75 years (range 22–59 years), and 65 age- and education-matched healthy control subjects, mean age 38.72 ± 6.89 years (range 23–59). All the participants were men. The participants were subdivided into 4 groups according to age: group 1 (20–29 years old), group 2 (30–39 years old), group 3 (40–49 years old) and group 4 (50–59 years old). For HIV-positive patients, there were 16 individuals in group 1, 17 in group 2, 17 in group 3 and 16 in group 4, and there were 17, 15, 16 and 17 individuals, respectively, for healthy people in these groups. Basic demographic data of the participants and clinical data of the HIV-positive subjects are summarized in **Table 1**.

**Table 1:** Basic demographic data on participants in the study.

Variable	Group	Number	Mean	*SD*	*p*
Age (years)	HIV + subjects	66	37.84	7.75	0.384
	Controls	65	38.72	6.89	
Education (years)	HIV + subjects	66	12.93	2.62	0.502
	Controls	65	13.07	3.33	
Gender (% males)	HIV + subjects	66	1	0.00	n/a
	Controls	65	1	0.00	
Current CD4 count (cells/mL)	HIV + subjects	66	688.72	285.87	n/a
Nadir CD4+ count (cells/mL)	HIV + subjects	66	112.23	67.79	n/a
Duration of cART	HIV + subjects	66	6.5	3.49	n/a
IHDS	HIV + subjects	66	11.25	0.50	0.684
	Controls	65	11.50	0.25	


*cART, combination antiretroviral therapy; IHDS, International HIV Dementia Scale; SD, standard deviation; n/a, not available.*

Inclusion criteria for healthy subjects were: (1) age over 18; (2) no signs of neurocognitive impairment on screening neuropsychological testing (International HIV Dementia Scale- IHDS); and (3) HIV sero-negative on polymerase chain reaction (PCR). HIV-positive subjects were included if they fulfilled following criteria: (1, 2, and 3) HIV sero-positive on PCR; (4) under the same cART regimen for at least 1 year. Exclusion criteria for both groups were: (1) active infiltrative or infective/opportunistic neurological illness; (2) acute or chronic ischemic lesions (including leukoaraoisis and lacunar strokes); (3) demyelinating disorders; (4) white matter hyperintensities (WMHs) in the region of interest; (5) history of head trauma: (6) chronic neuropsychatric illnesses; (7) history of drug dependence according to the Diagnostic and Statistical Manual of Mental Disorders, except for nicotine; (8) coinfection with hepatitis C virus and (9) contraindications for MR scanning.

Each participant underwent a complete screening neuropsychological testing for HIV dementia (International HIV Dementia Scale - IHDS). All HIV-positive subjects and healthy controls scored over 11, which classified them as ‘not eligible for further examination of possible dementia’ (Sacktor et al., 2005).

### MR Spectroscopy Protocol

All participants underwent structural MR imaging and intermediate echo-time 2D multi-voxel MRS on a 3T MR scanner (Trio Tim, Siemens, Erlangen, Germany). The protocol included a three-plane localizer, a sagittal 3D magnetization prepared rapid gradient echo (TR/TE, matrix size, duration), an axial fluid attenuation inversion recovery (FLAIR) sequence (5150 ms/105 ms, 256 × 220, 3:30 min) to rule out potential focal white matter lesions, a sagittal T1-weighted spin echo sequence (440 ms/3.8 ms, 320 × 320, 2:00 min) and a coronal T2-weighted turbo spin echo sequence (7150 ms/111 ms, 384 × 324, 2:17 min). Triplanar imaging was necessary to obtain adequate positioning of the multivoxel spectroscopic network.

**FIGURE 1:**
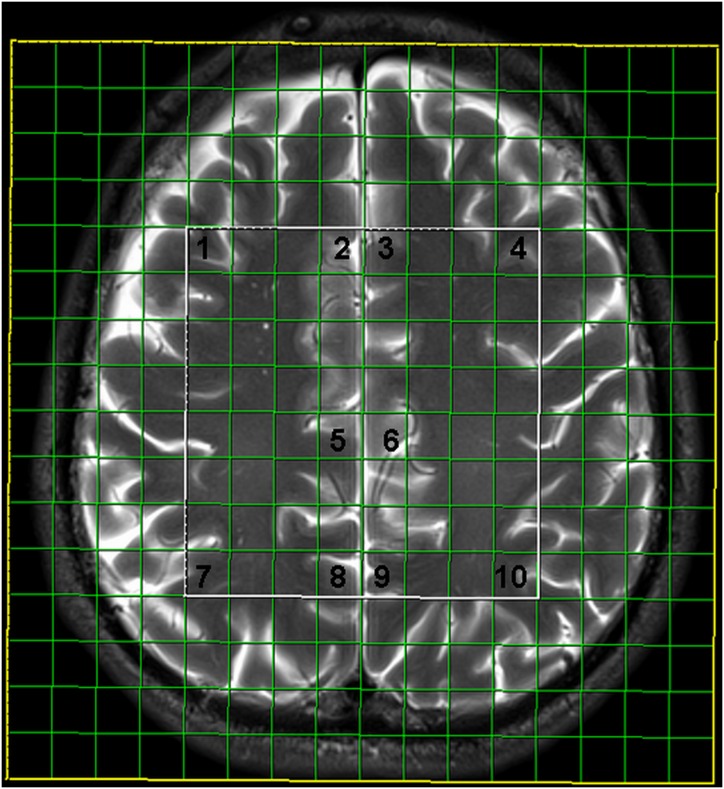
Multivoxel network with analyzed locations: 1- frontal cortex on the right, 2- ventral anterior cingulate gyrus on the right, 3- ventral anterior cingulate gyrus on the left, 4- frontal cortex on the left, 5- dorsal anterior cingulate gyrus on the right, 6- dorsal anterior cingulate gyrus on the left, 7- parietal cortex on the right, 8- posterior cingulate gyrus on the right, 9- posterior cingulate gyrus on the left, 10- parietal cortex on the left.

Two-dimensional intermediate echo time MRS was performed using point-resolved spectroscopy (TR/TE 1700/135 ms, scan time 8 min 17 s), with the following features: field of view (FOV) 160 × 160 × 10 mm; volume of interest (VOI) 80 × 80 × 10 mm, and thickness 10 mm. Interpolation resolution was 16 in all directions (right–left, anterior–posterior, foot–head). A weighted phase-encoding scheme was applied. External tissue signals were saturated with six manually positioned saturation regions. An automatic, volume-selective shimming method was used. A 64-voxel network was obtained, and 10 symmetric locations in the gray matter were examined, including the frontal and parietal cortices, the ventral and dorsal anterior cingulate gyrus, and the posterior cingulate gyrus (**Figure 1**). Two independent experienced neuroradiologists analyzed the spectra.

The spectra were later imported to a Siemens Leonardo workstation. Dedicated manufacturer’s software for MR spectroscopy was applied for baseline corrections, peak identification, and calculation of the ratios throughout the manually identified voxels. The intensity of NAA (at 2.02 ppm) and creatine (Cr) (at 3.0 ppm) peaks were measured, and the NAA/Cr ratio was calculated.

### Statistical Analysis

Statistical analysis was performed using SPSS software version 23.0 (Chicago, IL, United States). Descriptive statistics consisted of calculating mean values, standard deviation, median, interquartile range, maximum and minimum values. After confirming the necessary conditions, the univariate analysis of variance (ANOVA) test was performed to compare differences in NAA/Cr levels between HIV-positive subjects and healthy controls. After performing a correlation analysis and confirming a significant influence of aging on NAA/Cr level in both groups, a multivariate analysis, with age, education level and estimated duration of therapy added as covariates, was performed.

In order to minimize differences in NAA/Cr levels caused by an age effect, stratification of the sample was performed by dividing all participants into four age groups: group 1 (20–29 years old), group 2 (30–39 years old), group 3 (40–49 years old) and group 4 (50–59 years old). NAA/Cr ratios in the observed locations for correspondent age groups of HIV-positive and healthy controls were compared using ANOVA test. Relationship between age and NAA/Cr ratios were determined using linear regression model with interaction term (group × age). Correlations of NAA/Cr levels with educational level, duration of infection, and duration of cART were performed in all age groups of HIV-positive subjects using the Pearson’s correlation test. Statistical significance was set at a value of *p* < 0.05.

## Results

The comparison of NAA/Cr ratios of all HIV-positive subjects with all healthy controls showed a significant decrease in NAA/Cr levels in HIV-positive subjects in all locations. A multivariate analysis did not show any significant influence of educational level on NAA/Cr ratios in either of the groups The influence of infection duration on NAA/Cr levels was also not significant. Partial correlation of age and NAA/Cr levels showed a significant difference in majority of locations (5/10 measured locations) with the eta squared results ranging from 0.048 to 0.085. The presence of HIV infection showed a significant difference in all locations (10/10) with the eta squared ranging from 0.068 to 0.225. The results are summarized in **Table 2**.

**Table 2:** Results of the ANCOVA comparison of the NAA/Cr ratios in observed localizations between HIV-positive and control subjects with sequentially introduced age and educational level as covariates.

Localization	Group	*N*	Mean	*SD*	*p*	η^2^ (group)	η^2^ (age)	η^2^ (education)
Right frontal cortex	HIV + subjects	66	1.67	0.34	**<0.001**	0.214	0.050	ns
	Controls	65	2.06	0.29				
Right ventral ACG	HIV + subjects	66	1.37	0.25	**<0.001**	0.172	0.061	ns
	controls	65	1.63	0.21				
Left ventral ACG	HIV + subjects	66	2.15	0.34	**0.001**	0.102	0.048	ns
	controls	65	2.41	0.36				
Left frontal cortex	HIV + subjects	66	1.57	0.18	**<0.001**	0.171	0.074	ns
	controls	65	1.78	0.25				
Right dorsal ACG	HIV + subjects	66	1.93	0.30	**0.040**	0.074	ns	ns
	controls	65	2.08	0.26				
Left dorsal ACG	HIV + subjects	66	1.56	0.40	**<0.001**	0.225	ns	ns
	controls	65	2.04	0.35				
Right parietal cortex	HIV + subjects	66	1.97	0.35	**<0.001**	0.113	ns	ns
	Controls	65	2.25	0.32				
Right PCG	HIV + subjects	66	1.51	0.16	**<0.001**	0.218	0.085	ns
	Controls	65	1.77	0.28				
Left PCG	HIV + subjects	66	2.08	0.40	**<0.001**	0.195	ns	ns
	Controls	65	2.47	0.43				
Left parietal cortex	HIV + subjects	66	1.87	0.28	**0.008**	0.068	ns	ns
	Controls	65	2.02	0.32				


*η^*2*^ squared is considered significant when > 0.014. N, number; SD, standard deviation; ns, non significant; ACG, anterior cingulate gyrus; PCG, posterior cingulate gyrus. The numbers in bold indicate statistically significant values.*

**FIGURE 2:**
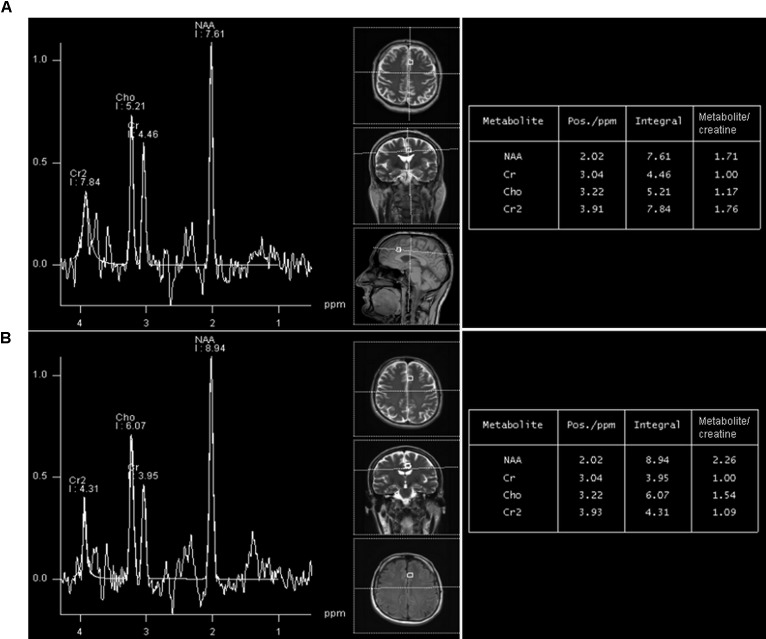
A representative long-echo MR spectrum located in the of HIV-positive subject **(A)** and age-matched healthy control **(B)**, from the group 20–29 years. NAA/Cr level in HIV-positive person is significantly lower (1.71) than in an age-matched healthy control (2.26).

When NAA/Cr ratios were compared in a stratified sample (age-matched groups), a significant decrease was observed in 8/10 locations in the group of the youngest HIV-positive subjects (20–29 years), compared to the correspondent age group of healthy controls (**Table 3**). In the 30–39 years-old groups, the differences were significant in 6/10 locations, and in the 40–49 years group, significant differences were observed in only 4/10 locations (**Table 4**). In the oldest group (50–59 years old), a significant decrease in NAA/Cr ratios was detected in HIV-positive subjects in only 1/10 locations (**Figure 2**).

**Table 3:** Results of the comparison of NAA/Cr ratios in observed locations between HIV-positive and control subjects in the age group of 20–29 years.

Localization	Group	*N*	Mean	*SD*	*p*
Right frontal cortex	Controls 20–29	16	2.23	0.24	**<0.001**
	HIV + 20–29	17	1.63	0.26	
Right ventral ACG	Controls 20–29	16	1.74	0.28	**0.016**
	HIV + 20–29	17	1.44	0.15	
Left ventral ACG	Controls 20–29	16	2.41	0.45	0.641
	HIV + 20–29	17	2.32	0.39	
Left frontal cortex	Controls 20–29	16	1.86	0.34	**0.037**
	HIV + 20–29	17	1.58	0.13	
Right dorsal ACG	Controls 20–29	16	2.21	0.49	0.136
	HIV + 20–29	17	1.92	0.24	
Left dorsal ACG	Controls 20–29	16	2.15	0.39	**0.014**
	HIV + 20–29	17	1.67	0.32	
Right parietal cortex	Controls 20–29	16	2.38	0.23	**<0.001**
	HIV + 20–29	17	1.92	0.19	
Right PCG	Controls 20–29	16	1.92	0.29	**0.003**
	HIV + 20–29	17	1.55	0.11	
Left PCG	Controls 20–29	16	2.48	0.63	**0.035**
	HIV + 20–29	17	1.95	0.27	
Left parietal cortex	Controls 20–29	16	2.11	0.24	0.107
	HIV + 20–29	17	1.93	0.19	


*N, number; SD, standard deviation; ACG, anterior cingulate gyrus; PCG, posterior cingulate gyrus. The numbers in bold indicate statistically significant values.*

**Table 4:** Results of the comparison of NAA/Cr ratios in observed locations between HIV-positive and control subjects in the age group of 50–59 years.

Localization	Group	*N*	*Mean*	*SD*	*p*
Right frontal cortex	Controls 50–59	16	1.74	0.09	0.320
	HIV + 50–59	17	1.58	0.29	
Right ventral ACG	Controls 50–59	16	1.46	0.08	0.334
	HIV + 50–59	17	1.30	0.31	
Left ventral ACG	Controls 50–59	16	2.44	0.24	**0.004**
	HIV + 50–59	17	2.01	0.20	
Left frontal cortex	Controls 50–59	16	1.66	0.18	0.104
	HIV + 50–59	17	1.49	0.18	
Right dorsal ACG	Controls 50–59	16	1.93	0.16	0.829
	HIV + 50–59	17	1.97	0.32	
Left dorsal ACG	Controls 50–59	16	1.80	0.26	0.425
	HIV + 50–59	17	1.59	0.47	
Right parietal cortex	Controls 50–59	16	2.12	0.38	0.282
	HIV + 50–59	17	1.92	0.28	
Right PCG	Controls 50–59	16	1.48	0.07	0.651
	HIV + 50–59	17	1.45	0.15	
Left PCG	Controls 50–59	16	2.42	0.17	0.326
	HIV + 50–59	17	2.18	0.46	
Left parietal cortex	Controls 50–59	16	1.64	0.22	0.098
	HIV + 50–59	17	1.99	0.37	


*N, number; SD, standard deviation; ACG, anterior cingulate gyrus; PCG, posterior cingulate gyrus. The numbers in bold indicate statistically significant values.*

A comparison of correspondent age-groups of HIV-positive subjects and healthy subjects with regard to educational level showed the following results:

(a) 20–29 years: the mean duration of education in the HIV-positive group was 15.2 years (range 10–16 years), and in the control group 15.38 (range 12–16 years); there was no significant difference between groups (*F* = 0.098, *p* = 0.758)
(b) 30–39 years: the mean duration of education in the HIV-positive group was 12.57 years (range 8–16 years), and in the control group 12.68 years (range 9–16 years); there was no significant difference between groups (*F* = 1.002, *p* = 0.701)
(c) 40–49 years: the mean duration of education for the HIV-positive subjects was 12.67 years (range 12–16), and in the control group 12.34 years (range 12–16), with no significant difference between groups (*F* = 1.098, *p* = 0.684)
(d) 50–59 years: the mean duration of education in HIV-positive subjects was 11.29 years (range 8–16), while in the control group it was 11.86 years (range 8–16), with no significant differences between groups (*F* = 1.123, *p* = 0.631).

There was no significant correlation of education level with NAA/Cr ratios in any of the observed age groups in HIV-positive subjects.

The estimated duration of HIV infection (based on self-report) was 4.4 years (range 3–8 years) in the youngest group of HIV-positive patients (20–29 years). For the group 30–39 years of age, the mean estimated duration of HIV infection was 8.57 years (4–14). Finally, in groups of HIV-positive patients 40–49 and 50–59 years of age, the estimated duration of HIV infection was 14 years (11–18) and 18.43 years (12–24), respectively. No significant correlation was found between NAA/Cr ratios and the estimated duration of infection in age groups of HIV-positive subjects.

Mean duration of cART for the whole sample of HIV-positive subjects was 6.5 years (range 1–16 years). For the group 20–29 years of age, the mean duration of ART was 2.23 ± 1.84 years (range 1–4 years), for the group 30–39 years of age, it was 6.82 ± 3.51 years (range 3–10), while in the 40–49 and 50–59 age groups, it was 11.21 ± 2.14 (range 8–14) and 14.54 ± 1.53 years (11–16), respectively. The resuts of correlation analysis between NAA/Cr ratios and duration of cART are demonstrated in **Table 3**. A significant positive correlation (the longer duration of cART, the higher NAA/Cr ratios) was most prominent in the oldest age group (50–59 years), observed in 8/10 voxels. In the youngest group (20–29 years old), there was no significant correlation (**Table 5**). Representative MR spectra from corresponding locations in HIV-positive person and age-matched healthy control are presented in **Figure 2**.

**Table 5:** The results of Pearson’s correlation test of NAA/Cr levels and duration of cART in observed locations according to the age groups in HIV-positive individuals.

Pearson’s correlation with duration of cART								
**Age**	**20–29**		**30–39**		**40–49**		**50–59**	
**Location**	***R***	***p***	***R***	***p***	***R***	***p***	***R***	***p***
Right FC	0.049	0.938	0.564	0.145	0.308	0.386	0.475	**0.013**
Right vACG	0.346	0.569	0.356	**0.043**	0.377	**0.048**	0.953	** < 0.001**
Left vACG	0.617	0.268	0.540	0.168	0.462	**0.015**	0.594	0.078
Left FC	0.425	0.295	0.544	0.105	0.625	0.054	0.428	**0.033**
Right dACG	0.367	0.543	0.574	0.082	0.453	**0.038**	0.474	**0.038**
Left dACG	0.354	0.559	0.436	0.24	0.517	**0.03**	0.539	**0.025**
Right PC	0.428	0.473	0.318	0.404	0.574	**0.082**	0.505	**0.024**
Left PC	0.351	0.562	0.303	0.428	0.625	**0.053**	0.463	0.112
Right PCG	0.713	0.177	0.687	**0.045**	0.523	**0.033**	0.391	**0.033**
Left PCG	0.268	0.663	0.623	**0.05**	0.508	**0.024**	0.456	**0.030**


*FC, frontal cortex; vACG, ventral anterior cingulate gyrus; dACG, dorsal anterior cingulate gyrus; PC, parietal cortex; PCG, posterior cingulate gyrus. The numbers in bold indicate statistically significant values.*

Correlating NAA/Cr dynamics in HIV-patients with age a significant increase was found when group 1 was compared with group 2 (regression slope + 0.1). Between the group 2 and 3 small decline in NAA/Cr was observed (regression slope -0.021). Another increase was observed when groups 3 and 4 were compared (regression slope + 0.0053). Regression slope for healthy individulas showed minimal but continous decrease (regression slope -0.03).

## Discussion

There is an ongoing debate about the aging pattern in chronic HIV infection. In the past decade, a significant amount of data on the HIV-related aging process has been collected, based predominantly on neuropsychological assessment (Shouten et al., 2016). The effect of the physiological aging of the brain on cognitive changes in chronic HIV infection has been studied recently, using both neuroimaging methods and neurocognitive testing. Published neuroimaging studies rely mostly on volumetry and DTI, designed to evaluate cortical cerebral atrophy and the diffusivity changes in white matter (Ances et al., 2012). It is a known fact that HIV infection is associated with greater than chronological age-related atrophic changes, that are pronounced in the frontal and temporal regions, the brainstem and the basal ganglia (Chang et al., 2011; Sanford et al., 2017). Although DTI demonstrated mainly normal age-related changes in mean diffusivity, elevated diffusion and lower fractional anisotropy were observed in younger HIV-positive individuals, as compared to healthy subjects. This result was explained as attributable to the presence of ongoing neuroinflammation in younger subjects with short-time cART (Chang et al., 2008).

There are only a few single-voxel spectroscopic studies that have examined age-dependent changes in brain metabolite ratios in HIV-positive individuals in the era of cART, which reported showing signs of premature aging process (Chang et al., 2008; Cysique et al., 2013; Sailasuta et al., 2016). Although in our study, the levels of choline (Cho) and myoinositol (mI) were also measured, we failed to present significant age-related differences in Cho/Cr and mI/Cr. This finding is concordant with the study of Eylers (Eylers et al., 2016), who found no significant age-related chanegs in these two metabolite ratios in healthy population, either. The explanation for the lack of significant differences might lie in the good control of HIV infection in observed patients (HIV- seronegative state), competent immune system and the absence of manifest neurocognitive disorder, but also in the small study sample. An age-related decrease in NAA/Cr was observed mainly in the group of HIV-positive subjects with manifest neurocognitive disorders.

When comparing HIV-positive patients of different age groups with age-matched healthy subjects, the results of our study showed that NAA/Cr ratios were significantly decreased in chronically infected, virally suppressed HIV-positive subjects, compared to healthy controls. This has also been observed in previous spectrosopic studies (Bairwa et al., 2016; Boban et al., 2017). In HIV-positive subjects, the presence of HIV infection was the strongest contributor to this decline, followed by age. The educational level did not influence the NAA/Cr decline in either groups.

Based on the results of several neuropsychological studies in HIV-patients with manifest neurocognitive impairment, the hypothesis of accelerated aging in chronic HIV infection has been proposed (Shouten et al., 2011, 2016). The term ‘accelerated aging’ represents the interaction of HIV infection and physiological aging that progressively increases the risk of age-related comorbidities (Cohen and Torres, 2017).

However, the latest neuropsychological and volumetric studies have proposed a new hypothesis. Cole et al. (2017) failed to show the relationship between atrophy scores and chronological age and duration of HIV infection, which led to the conclusion that the aging process in chronic HIV infection is actually accentuated (Cole et al., 2017). In ‘accentuated aging,’ the initial infection process triggers neuronal damage; however, the burden remains static over time under cART. This was confirmed in a volumetric study where there were no signs of progressive atrophy in neurologically asymptomatic HIV-positive subjects in a 2-year follow up study, although the baseline volumes of the brain regions were decreased compared to age-matched HIV-negative controls (Sanford et al., 2018). Additionally, a more recent comprehensive and longitudinal study of Cole, found no evidence for accelerated brain aging or progressive cognitive decline in HIV-positive subjects, over 2 years, using various neuroimaging data (Cole et al., 2018).

In our cohort, lower NAA/Cr ratios were observed in all measured locations when young HIV-positive patients were compared with age-matched healthy subjects. However, in the oldest age groups (50–59 years old) of HIV-positive and healthy subjects, there was no significant difference in NAA/Cr ratios, except in one location.

**FIGURE 3:**
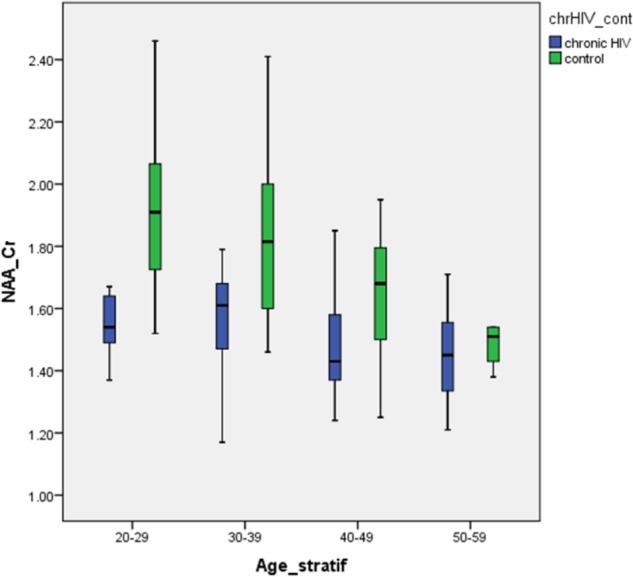
Differences in the NAA/Cr ratios between HIV-positive patients and healthy controls in analyzed locations, according to age group.

The prominent decrease in NAA/Cr ratios in the youngest HIV-positive subjects and the absence of significant differences between HIV-positive and healthy subjects in the oldest age groups is concordant with the hypothesis of initial damage to the neurons that occurs early in the course of HIV infection. Subsequently, the burden of risk to neurons remains static under highly penetrant cART. Furthermore, when linear age regression analysis was performed in HIV-positive groups, after an initial significant decrease in NAA/Cr, a steep increase was observed, indicating partial recovery after initiation of cART (**Figure 3**). A slight decrease in the group 3 (40–49 years) was present, although not reaching low levels measured initially (group 1), most probably due to the relatively small sample. Looking at all HIV-positive age groups, **Figure 3** shows a clear initial drop in NAA/Cr with slow but steady increase and finally almost reaching the same level as in healthy subjects. In other words, our results confirm the second hypothesis, that the aging process in chronic, well-controled HIV-infection is accentuated, rather than accelerated. The results of the MR spectroscopic study are in concordance with the results of the latest volumetric and neuropsychological studies (Cole et al., 2018; Sanford et al., 2018).

Previous studies have suggested a positive effect of therapy in controlling the ongoing brain inflammation and consequently slowing down the process of neurodegeneration (Chang et al., 2008; Cysique et al., 2013; Sailasuta et al., 2016). Significant positive correlations between NAA/Cr ratios and the duration of cART were observed in the age group 50–59 years (who were under cART for the longest time), while in the group 20–29 years old, no significant correlations were observed (the shortest mean duration of therapy). This finding supports the beneficial effect of early and long standing cART. At present, the introduction of cART starts immediately after confirmation of HIV-seropositive status, regardless of current immnological parameters, thus enabling the timely control over the process of neuroinflammation and subsequent neurodegeneration. Evenmore, the fact that we found an increase in the NAA/Cr in our cohort of neurologically asymptomatic, virally suppressed HIV-positive individuals under cART, suggests not only the accentuated brain aging in HIV-patients under cART but also a possibility of neuronal recovery after an initial brain damage. A recent MR spectroscopic study (Bladowska et al., 2013) showed a significant decrease in the NAA/Cr ratio in the gray matter of posterior and anterior cingulate gyrus, and in the parietal white matter in patients receiving cART, as compared to the healthy controls. The advanced HIV infection and high pre-cART viral load were suggested to contribute to the initial brain injury. This result is especially important in the light of current European AIDS Clinical Society (EACS) guidelines that recommend the introduction of cART immediately after the confirmation of HIV-seropositive status, regardless of current immunological parameters (Ryom et al., 2016), contrary to the prior EACS guidelines that recommended commencing cART when CD4+ T cell counts were only below 350/μL (Clumeck et al., 2008).

One important limitation of the study is is the cross-sectional design, which did not allow the examination of the possible process of accelerated aging over time. Individual trajectories of brain aging could be assessed only in the setting of a longitudinal study. The other limitation of the study is the inclusion of male subjects only. However, since our HIV-positive study population consisted mainly of MSM population, randomizing of the healthy control group was directed in the same way. Nevertheless, based on the data from the literature (Chiu et al., 2014), there were no significant gender-related differences in NAA/Cr ratios in selected regions (anterior and posterior cingulate cortices) during healthy brain aging.

## Conclusion

Based on the results of this multi-voxel MR spectroscopic study, the aging pattern in chronic HIV infection under long standing cART is accentuated rather than accelerated. This is in concordance with the results of previous neurocognitive and volumetric studies. MR spectroscopy is the third method confirming the hypothesis of accentuated brain aging in chronic, virally supressed and asymptomatic HIV-positive patients. It seems that the initial HIV-related neuronal damage causes a significant decline in the NAA/Cr ratios in the supratentorial gray matter. After the initiation of cART, the NAA/Cr ratios increase continuously and become almost equal to healthy aging individuals. Significant positive correlations between the cART duration and the level of neurodegeneration seem to confirm the beneficial effect of early cART initiation in controlling the inflammation and neurodegeneration. The increase of NAA/Cr soon after the initiation of cART in our cohort sugests at least partial recovery of neuronal function.

## Ethics Statement

This study was carried out in accordance with the recommendations of Ethical Committee of Faculty of Medicine, University of Novi Sad. The protocol was approved by the Ethical Committee of Faculty of Medicine, University of Novi Sad. All subjects gave written informed consent in accordance with the Declaration of Helsinki.

## Author Contributions

JB, MT, and DK: had full access to all the data in the study and take responsibility for data integrity and accuracy of analysis. JB, DK, and MT: study concept and design. All authors: acquisition, analysis, or interpretation of the data. JB: drafting of the manuscript. MT, DK, SB, and JB: critical revision of the manuscript for important intellectual content. DL: statistical analysis. JB, MT, and DK: administrative, technical, or material support. MT and DK: study supervision.

## Conflict of Interest Statement

The authors declare that the research was conducted in the absence of any commercial or financial relationships that could be construed as a potential conflict of interest.
